# Testing Antigens, Antibodies, and Immune Cells in COVID-19 as a Public Health Topic—Experience and Outlines

**DOI:** 10.3390/ijerph182413173

**Published:** 2021-12-14

**Authors:** Monica Neagu, Carolina Constantin, Mihaela Surcel

**Affiliations:** 1 Immunology Laboratory, Victor Babes National Institute of Pathology, 99-101 Splaiul Independentei, 050096 Bucharest, Romania; neagu.monica@gmail.com (M.N.); msurcel2002@yahoo.com (M.S.); 2 Pathology Department, Colentina University Hospital, 19-21 Șoseaua Ștefan cel Mare, 020125 Bucharest, Romania; 3 Doctoral School of Biology, Faculty of Biology, University of Bucharest, 91-93 Splaiul Independentei, 050095 Bucharest, Romania

**Keywords:** SARS-CoV-2, methodology, detection

## Abstract

The current COVID-19 pandemic has triggered an accelerated pace in all research domains, including reliable diagnostics methodology. Molecular diagnostics of the virus and its presence in biological samples relies on the RT-PCR method, the most used and validated worldwide. Nonconventional tests with improved parameters that are in the development stages will be presented, such as droplet digital PCR or CRISPR-based assays. These molecular tests were followed by rapid antigen testing along with the development of antibody tests, whether based on ELISA platform or on a chemiluminescent microparticle immunoassay. Less-conventional methods of testing antibodies (e.g., lateral flow immunoassay) are presented as well. Left somewhere in the backstage of COVID-19 research, immune cells and, furthermore, immune memory cells, are gaining the spotlight, more so in the vaccination context. Recently, methodologies using flow-cytometry evaluate circulating immune cells in infected/recovered patients. The appearance of new virus variants has triggered a surge for tests improvement. As the pandemic has entered an ongoing or postvaccination era, all methodologies that are used to monitor public health focus on diagnostic strategies and this review points out where gaps should be filled in both clinical and research settings.

## 1. Introduction

As the outbreak of the coronavirus disease 2019 (COVID-19) has gathered, over one year, valuable information in both research and clinical areas, we need to use this informational asset to further control this infection and move toward its annihilation. In this epic battle, human versus virus, epidemiological data reside and depend on the accessibility and “spreadability” of molecular testing. Within the area of molecular diagnosis, there are several issues that testing should overcome. First, SARS-CoV-2 has an identity with SARS-CoV and MERS-CoV because SARS-CoV-2 is the result of mutations leading to a new strain. Furthermore, the strain has its own genetic evolution and, as we have already witnessed since the beginning of 2020, this evolutionary process is ongoing. In this light, molecular diagnosis should be thoroughly investigating this genetic evolution.

In the diagnosis domain of this infectious disease, the immune response characteristics evaluation is a seminal issue [1]. A physiological immune response raised to an infection leads to pathogen elimination via innate and adaptive immune response. A proper immune response would repair the damaged tissue and would further induce the generation of memory-specific immune cells. The later cells would be reactivated upon a second encounter with the same pathogen. There are still issues that must be clarified using various investigation methods, in both infected patients as well as in vaccinated subjects. Therefore, we are still gathering knowledge regarding antibody persistence, their protective effect, and whether there is cross-reactivity with antibodies raised against other Coronaviridae. Inflammatory response triggered by a hyperactivation of immune components, mainly in severe infection cases, still lacks information and this issue is important in the search of criteria to stratify patients that are difficult to treat. Last, but not least within the immune response, immunological memory type, its persistence, and efficacy in both infected as well as vaccinated subjects are still a matter of intense research [2].

Finally, all these equally important issues in the current pandemics rely on standardized, reliable methods that the current review is outlining [1].

## 2. Technologies to Assess Specific Antigens

Laboratory diagnosis in COVID-19 is influential in combating the spreading of SARS-CoV-2 infection. Moreover, laboratory tests dictate the clinical decisions regarding the infected patient. These tests comprise the ones that detect the viral genome and testes that detect the viral proteome. Upon molecular and antigen tests, patients were classified as positive or negative for the presence of SARS-CoV-2. Nevertheless, all tests have two seminal characteristics/parameters, namely, percent positive agreement (PPA), describing the actual sensitivity of the test, and percent negative agreement (PNA), describing the specificity of the test [3].

In diagnosing SARS-CoV-2 infection, the most widely used test is the molecular testing. Real-time reverse transcription polymerase chain reaction (RT-PCR) is the most well-known and extensively used molecular analysis. The test relies on nucleic acid amplification and detects unique sequences of SARS-CoV-2 [4]. The other type of test, the antigen tests, can detect the presence of SARS-CoV-2 without amplifying viral components, but these tests are less sensitive than the molecular ones. Commonly, any negative antigen test is confirmed with a molecular test so that the patient can be declared negative for COVID-19. Both molecular and antigen tests would detect patients in the acute phase of infection [5,6].

Molecular tests can be performed on various samples such as nasopharyngeal swab, lower respiratory system samples, sputum, tracheal aspirate, capillary blood, serum, and plasma. The use of a variety of samples leads to various performances of the tests.

False positivity in RT-PCR tests was reported and it has several explanations. A recently found explanation of false-positivity can be due to a newly reported mechanism in which SARS-CoV-2 RNAs can be reverse-transcribed and hence integrated in the human genome. Therefore, this transcription of the integrated sequences can give PCR-positive results. The authors found chimeric transcripts made of virus fused to cellular sequences in primary cells of patients [7].

### 2.1. Quantitative Real-Time Reverse Transcriptase-PCR

RT-PCR is a technology used on a large scale for diagnosing different viral infections, such as Ebola and Zika infection. Therefore, when this new coronavirus infection hit the world, the already used technology expanded for this virus.

Viral RNA is detected using RT-PCR, and the test reports the abundance of viral genetic material, with results being reported as “qualitative”. In this test, the abundance detected above an established threshold gives the already famous “positive” results. Establishing the appropriate threshold is probably the most argued issue in this pandemic. The main purpose of the imposed threshold is not to miss false negative results while minimizing false positives [8].

In a nutshell, the technology consists of two clear stages: viral RNA is reverse transcribed into DNA, that is amplified due to polymerase chain reaction (PCR) [9]. FDA and the Centre for Disease Control and Prevention (CDC) recommended, for this test, several regions to be detected: viral nucleocapsid N1, N2, and human RNase P gene [9]. The World Health Organization (WHO) has recommended detecting CoV-2 RNA-dependent RNA polymerase (RdRP) and envelope (E) genes [10].

An infectious virus particle has intact nucleic acid covered by a capsid. Starting from this assertion, there are several points that need more attention. Hence, RT-PCR detects viral RNA, but this genetic material is not mandatorily appended to a replicating virus [11,12]. There are studies that show the relation between a cultivable virus and the viral RNA that is shed. Moreover, recovering patients, although not infectious anymore, are still shedding viral RNA [13]. When sampling a nasopharyngeal swab, proteins and debris are eliminated and the extricated RNA is tested, but this RNA contains both individual and viral RNA. Hence the entire extracted RNA is reverse transcribed into DNA and amplified by PCR. For major viral RNA loads, the reverse-transcribed DNA will have mainly viral genetic information, but samples with borderline viral load will not have such clear results [13,14].

RT–PCR is an end-point technology; it will not give information on past infection, information needed for registering epidemiological events. The only test that can directly indicate if the infectious viral particles are still present is the viral cultivation in particular cells, such as African green monkey kidney Vero C1008 clone E6 cells [15].

Viral load in an individual is associated with the severity of the infection, but this issue has also things that should be clarified [16]. Studies regarding the relation between viral load and clinical evolution are still very few, the retrospective nature of the investigation is limited, and sample sizes and selection bias are still not relevant. Another issue that is raised in this field is the PCR type used to measure the viral infection. Sample type, patient’s age and gender, comorbidities, and probably many more factors influence the viral load, parameters that are still to be established [17] along with the viability and infectiousness of the virus [18]. All these parameters should be related to the results that RT-PCR test would provide.

### 2.2. Nonconventional Tests—Droplet-Digital PCR

As current diagnostic tests are based on the RT-qPCR method, several limitations in terms of sensitivity and quantification have emerged. To improve its performance, new, improved tests are developed. Thus, in a study, published in March 2021, qPCR and droplet digital PCR (ddPCR) were tested for their capability to detect low amounts of viral RNA. ddPCR is a highly sensitive technology that uses a water–oil emulsion droplet system so that nucleic acid samples are partitioned in 20,000 nanoliter-sized droplets serving as independent test tubes [19]. Each sample would have thousands of individual partitions, with or without template DNA [20]. A PCR reaction develops in each tube and is examined for amplified target DNA by fluorescence [21]. The limit of detection of ddPCR is about 0.005%, much lower when compared to that of RT-PCR (1%), pyrosequencing (5%), melting curve analysis (10%), and Sanger sequencing (20%) [22]. This technology can overpass RT-PCR because it brings absolute quantification of DNA copies without using external calibration curves. The test bypasses known PCR inhibitors and hence provides higher accuracy, reproducibility, and increased sensitivity, especially for low concentrations of the searched molecules or degraded samples [20,23].

When directly comparing ddPCR with RT-PCR, the cycle threshold (CT) of the viral RNA identified by RT-PCR significantly varied related to the sequences of the primer and probe sets, while the copy number of the viral RNA depicted by ddPCR was effectively quantified with in vitro transcript RNA, cultured viral RNA, and RNA from clinical samples. Authors conclude that ddPCR could be used as an extremely sensitive and compatible diagnostic method for viral RNA detection [24]. Another group has developed a multiplex ddPCR for sensitive quantification of specific RNA with respect to human-derived RNA in screening and monitoring COVID-19 patients. This multiplex ddPCR detects, simultaneously, SARS-CoV-2 E, RdRp, and N viral RNA, and human Rpp30 DNA and GUSB mRNA (internal nucleic acid extraction and control). De Kock et al. proved that RT-ddPCR assay sensitivity was not affected by the total nucleic acids background. This is not the case for classical standard RT-PCR because total nucleic acids affect sensitivity [25]. Another multiplex ddPCR analysis was tested by Deiana et al. Comparing swabs with or without RNA extraction, the group has shown that the direct approach generated equal RNA copies in comparison to the extracted ones. Therefore, using ddPCR direct quantitation of virus SARS-CoV-2 in nasopharyngeal swab yielded an efficient quantitation [26]. Molecular analysis performed in patients’ plasma using ddPCR in comparison to classical PCR has shown also encouraging results. In plasma harvested from COVID-19 patients diagnosed in mild, moderate, and critical disease, virus was detected in 91% of patients when using ddPCR and in 87% when using RT-PCR. Both methods could detect RNAemia with ICU patients having the highest prevalence [27].

However, despite its high sensitivity, high specificity, and its potential clinical utility, the ddPCR approach implies high financial resources and highly trained personnel, making this powerful method still unaffordable for low-income countries.

### 2.3. Antigen Detection Tests

Another SARS-Cov-2 test method for diagnosing an active case is the antigen test, useful in early stages of the infection. The tests can detect viral antigen in the nasal, oral, and respiratory tract, sites in which the virus is actively shed, and hence has the highest infectivity.

These tests can detect viral presence up to 2 days before the onset of symptoms and are easy to perform. As specific antibodies are detectable at the earliest within the first week from the symptom onset, antigen tests can detect early infection. The test results depend on the duration of viral shedding and on several clinical parameters, such as disease severity, duration of the illness, and patient’s immune response. Viral shedding becomes undetectable around one month after symptoms onset or much earlier when the symptoms disappear.

Antigen tests can identify nucleocapsid (N) or spike (S) proteins [9]. In antigen testing, N is a good target because it is a conserved and abundant antigen. The antigen tests can have enzyme-linked immunosorbent assay (ELISA) format or a lateral flow rapid-test format. The ELISA format relies on the existence of a pair of specific antibodies that would recognize the target antigen, the tests having high sensitivity and specificity.

The ELISA format gives accurate results but needs above-average equipment and staff to handle the methodology, these issues being somewhat limiting in less-developed laboratories.

The lateral flow format or antigen rapid test can be used in a general screening of a population and can be handled even by nonmedical laboratories. It resembles HIV rapid test using serum, plasma, or fingertip blood. The rapid test gives visually interpretable results in around 20 min, being a point-of-care (POC) setting.

Supplementary tests that aid the clinical management of the infected patients are currently used in several units; these comprise coagulation tests, indicators of cytokine storm (e.g., interleukin-6), ferritin, granulocyte colony-stimulating factor (G-CSF), macrophage inflammatory protein–1α (MIP-1α), and tumor necrosis factor–α (TNF-α) [28].

**Figure 1:**
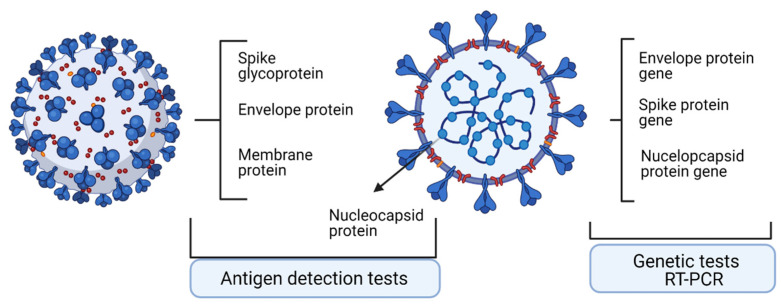
Main molecular targets and antigens detected in SARS-Cov2 infection used in diagnosis. Created with BioRender.com. (access on 1 October 2021).

A summary table of genome and proteome testing in viral infection [3] is presented in Table 1, and a schematic outline of the tests is shown in Figure 1.

## 3. Technologies to Assess Specific Antibodies

### 3.1. Antibody Dynamics in Infection

In SARS-CoV-2 infection, the first antibody that appears is IgM and it can be depicted from the fourth day of infection. IgM will increase until the 20th day when a peak is established and would diminish gradually while IgG appears. IgG will start to appear from the seventh day, peaks on the twenty-fifth day, and maintains its level one month after infection [29]. Seroconversion (specific IgG or IgM antibodies detection) occurs almost simultaneously or sequentially. Their values after 6 days of detection (after seroconversion) would reach a plateau concentration and will no longer vary [30]. Vaccination has the same dynamics in antibodies as the infection [31,32].

Patients with mild and severe forms display, over time, a strange increase in IgM titers [33]. It was shown in the severe group, compared to the nonsevere group, that IgG and IgM titers are high, probably as a consequence of the polyclonal stimulation caused by the infection. Patients with severe disease have a high IgG response but mild cases will develop a faster peak IgM response [34,35].

In asymptomatic patients, or better named oligosymptomatic patients, antibodies are depicted but their titers are not as high as the ones detected in symptomatic patients. We have also found, in accordance with other groups, that oligosymptomatic patients can display a fading of the IgG, in quite a high proportion [36] (40.0%, compared to almost 13% in symptomatic patients) [30].

In a recently published study, it was shown that another Ig is entering the spot line. IgA represents the most abundant antibody class produced in humans that is critical in the first line of antimicrobial/antiviral defense; it is a special antibody that patrols the mucosal boundaries [36]. Hence, we have shown that IgA in infected subjects has high levels and follows the IgG dynamics, levels that are detectable even after 8 months postinfection [37]. Because it neutralizes pathogens, IgA should also enter the panel of tested antibodies in COVID-19 [37].

Some points regarding antibodies as a general term and the neutralizing capacity should be detailed. Neutralizing antibodies (NAbs) display a clear protection capability in various viral/bacterial infections and can be generated through two main pathways: upon the actual infection and/or upon artificially induced immunization. An NAb would physically stop the pathogen from entering the target cells and hence would hinder the subsequent spreading of the infection [38]. Furthermore, NAbs would sterically change the normal conformational properties of the virus, and therefore impede, once more, its entrance to the target cell. In passive immunization, when NAbs from convalescent plasma are used, the main property of NAbs (neutralizing capacity) will clinically help patients still fighting the infection. Although it has a transient effect, it will help the patient to recover until its own NAbs are produced in sufficient quantities [39]. Another type of neutralizing antibodies that do not address the viral particle are the ones that hinder the receptors on the target cells and block the virus entry. Even though it is a neutralizing mechanism, it is termed as an infection-blocking mechanism. Several monoclonal antibodies against the SPIKE protein of SARS-CoV-2 have been either isolated from convalescent plasmas or designed and expressed de novo in the laboratories. This last type of therapy was attempted to artificially develop neutralizing antibodies that would inhibit virus infection; nevertheless, the results are not proven satisfactory yet. For example, Shi et al. identified two mAbs (CA1 and CB6) from COVID-19 patients, able to block the SARS-CoV-2 RBD binding to their target receptor ACE-2 [40].

**Figure 2:**
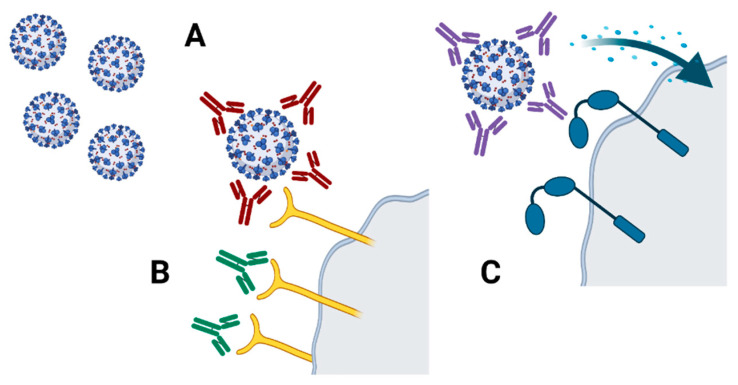
Generated antibody types during viral infections. (**A**) Neutralizing antibodies can link to the viral particle, hindering its entrance in the target cell. (**B**) Antibodies that can link to the specific receptor that is used by the viral particle, hindering its entrance in the target cell. (**C**) Low-affinity antibodies linked to the viral particle that can activate Fc-receptor on the target cell and thus favor viral entry into the cell generating ADE-related mechanisms. Created with BioRender.com. (access on 15 September 2021).

Figure 2 shows a scheme representing NAbs and their action mechanism. Moreover, low-affinity Abs that can lead to antibody-dependent enhancement (ADE) in viral infection are important players in the immune response and deserve further investigation. Knowledge gathered during SARS and MERS-CoV infection has shown that pre-existing, nonneutralizing, or poorly neutralizing antibodies that are generated during natural or artificial immunization can lead to ADE. As immunotherapy and vaccine are clinically applied, ADE is to be taken into consideration. ADE reduction should be taken into consideration in COVID-19 as a full-length, protein-based approach can lead to ADE phenomenon as previously reported in MERS-CoV [41].

### 3.2. Technologies to Assess Antibodies

Along with SARS-CoV-2, six other coronaviruses (OC43, 229E, SARS-CoV, NL63, HKU1, and MERS-CoV) were found as infectious agents in humans [42,43,44,45,46], and this is important when assessing test’s specificity. Among all these coronaviruses, the highest chance that antibodies generated in the current pandemic to cross-react is with SARS-CoV because of their close phylogenetic affiliation, high genome, and protein sequence’s identity [47]. This cross-reactivity within antibodies pattern is important in regions/countries affected by both epidemics [48]. Antibodies raised against major structural proteins of SARS-CoV2 are those against N and S proteins [46]. In a recent study using several testing platforms, it was shown that receptor-binding domain (RBD) protein ensures the best specificity, while N protein is endowed with high cross-reactivity between SARS-CoV and SARS-CoV-2 [49]. Capture ELISA has proven the best technical performance, and N-specific antibodies tested in SARS-CoV former patients disappeared faster than RBD-specific antibodies [50].

Serological (immunoassays) are developed for qualitative or quantitative investigation of SARS-CoV-2 antigens and/or anti-SARS-CoV-2 antibodies. In general, there are ELISA-based assays in various versions, indirect immunofluorescence assays, lateral flow immunoassays, and neutralization tests. Immunoassays are continuously developing in this new coronavirus infection [51].

Rapid tests with a sensitivity of 88.66% and specificity of 90.63% for both IgG-IgM specific for SARS-CoV-2 can give results in 15 min [52]. Tests such as these performed in extralaboratory environments have several disadvantages. Sampling and handling performed by nonlaboratory personnel could seriously affect the results. Moreover, if the infected person is within the first week of contact, antibodies could have not been produced yet [53]. Thus, the false negativity of the result would clear a probable infection-spreading person. The low sensitivity would yet again give false negative results, as it cannot detect intrinsic immunological differences and immune responses between individuals. There is an overabundance of antibody tests for SARS-CoV-2 having sensitivities from 45 to 100%, and specificities from 96 to 100%, as presented by the Foundation for Innovative New Diagnostics [51].

There are several technologies that are implemented in laboratory use and choosing one or the other or several ones is dictated by their speed of results, affordability, accuracy, standardization, wide implementation, and so forth.

Luciferase immunoprecipitation test (LIPS) is a very rapid serology test developed generally ten years ago [54,55]; the test has good sensitivity, but low specificity. The low specificity is due to the fact that antibodies generated in SARS-Cov2 infection and prior SARS epidemy are cross-reactive. This cross-reactivity is not unexpected, as N proteins from both viruses are highly related. Recently, LIPS was used to detect human infection by bat orthoreovirus [56]. Therefore, LIPS can be further developed as a rapid detection tool in COVID-19. Indirect ELISA is a medium time serology assay. When assessing indirect ELISA for antibodies raised against SARS-CoV-2, several conclusions were drawn. The published data show that IgG binding is specific and sensitive, but the same characteristic cannot be displayed for IgM antibodies. Finding that was proved, whether the sera was depleted for IgG or not [50]. Multiplex Luminex tests are usually used in the biomarkers screening stage. In this infection, the test had very good performance for six recombinant proteins. The downfall of the test is that not all the current laboratories have this technology and have the specific know-how implemented. The obtained results for IgG have similar performance to indirect ELISA [50]. Capture ELISA is also a medium time serology assay. Recently, it was shown that when using a horseradish-conjugated RBD protein (HRP-RBD), a significant improvement of IgM detection was obtained. The improvement outperforms indirect ELISA’s performance, achieving a perfect 100% specificity and sensitivity of 96% for all PCR-positive patients [50].

Rapid diagnostic tests (RDTs) aim to combine antigen and antibody tests for the rapid diagnostics of the infection. In a recent study, this diagnosis strategy performance was assessed, combining antigen testing with IgM or IgG RDTs. It was reported that the combination of Ag and IgM/IgG RDTs detected up to 84.0% of COVID-19 confirmed cases at admission. As antigen and antibodies’ RDTs showed low performances when used individually, their combination greatly improves their potency to identify most COVID-19 patients [57].

## 4. Technologies to Assess Specific Immune Cells

### 4.1. Immune Cell Response upon Infection

**Figure 3:**
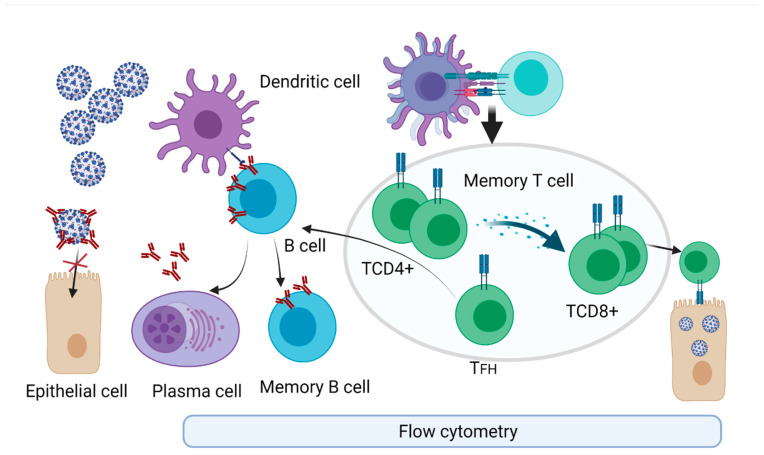
Cellular immune response upon infection detected using flow cytometry. Activated DCs present antigen and co-stimulatory molecules to specific naïve T cells, which become activated and further differentiate into effector cells (T CD8+ cytotoxic cells), T helper cells (CD4+). Activated DCs can directly activate specific B cells and induce B cell differentiation. T follicular helper (TFH) cells help B cells to differentiate into plasma cells that secrete specific antibodies and further generate B memory cells. Generated antibodies physically hinder the entrance of new viral particles into cells. Memory T cells are generated to sustain the long-time cellular immune memory, and CD4+ T cells memory cells secrete cytokines that induce the cytotoxic activity of T cells CD8+ that attack infected cells, stopping the further viral reproduction. T and B memory cells sustain long-lasting memory of the infection, and upon a second encounter with the same virus, quickly trigger all the necessary immune pathways. Created with BioRender.com. (access on 15 September 2021).

There is a complex cellular machinery that is triggered upon infection. SARS-CoV-2 would attack epithelial cells in the nasal cavity through binding to ACE2 receptors [58]. Once the virus has entered the cells, activation of transcription factors, IRF3/7, and NF-κB are triggered, and type I interferons (IFNs) and inflammatory cytokines are produced [59]. Generated IFNs would activate adaptive immune cells and would prevent viral spread by stimulating other immune cells that start to produce various inflammatory cytokines, chemokines, and antiviral enzymes [60,61]. Similar to SARS-CoV and MERS-CoV [62], SARS-CoV-2 can evade type I IFNs and can suppress IFN induction and intracellular signaling [63,64,65]. Alveolar macrophages would be the first immune cells that respond to the viral attack. Through their complex array of inflammatory-related secreted molecules and receptors (TLR2, 4, 6, IL-1R, IFNγ-R, TNFR, TREM2, IL-10R, TGFBR), these cells enter the immune network, controlling immune response [66]. Specific dendritic cells (DCs), residents in the attacked tissues, having a CD103+ phenotype permeate through the epithelial layer and capture and present specific antigens [67]. Viral antigens are processed by DCs and presented in MHC I and MHC II restriction to CD8+ T and CD4+ T cells, respectively [68]. Helper CD4+ T cells stimulate B cells and CD8+ T cells, and help the induction of memory cell populations [69]. In the meantime, DCs migrate to the lymph nodes to educate naïve T cells [70]. In viral infections, Th1-type immune response is dominant in the generated adaptive immune response [71]. Th1 CD4+ T cells, when activated, secrete GM-CSF, further activating inflammatory monocytes CD14+ CD16+ that generate IL-6, an inflammatory cytokine [72]. Another Th subpopulation, Th17, produces IL-17, hence recruiting additional monocytes, macrophages, and neutrophils that generate mainly IL-1β, IL-6, and IL-1, highly potent proinflammatory cytokines [73]. An outline of the main cellular network generated in the SARS-CoV-2 infection is presented in Figure 3.

In terms of technologies that can evaluate the proportion of circulating or resident lymphocytes, flow cytometry, with all its versions, is, for now, the best method. Therefore, using multiparametric flow cytometry whole peripheral blood samples harvested from COVID-19 convalescent patients showed clear immune cells differences compared to controls. Even after 3 months postinfection, patients had activated cytotoxic CD8+ T cells with high HLA-DR and CD38 expression. Patients had CD3+ CD4+ and CD3+ CD8+ effector memory higher than normal, while CD25+ Foxp3+ T regulatory cells were lower. Transitional B cell and plasmablast levels were significantly elevated, while innate immunity cells, such as neutrophils, were low [74]. Similarly, flow cytometry was used to evaluate the immune response of patients with acute respiratory distress syndrome (ARDS) associated with COVID-19. Immature neutrophils were found in both blood and bronchoalveolar lavage, accompanied by CD4+ and CD8+ T-cell lymphopenia. Tregs cells and Th17 cells were found in higher proportions in the bronchoalveolar lavage. CD4+, CD8+ T cells, and macrophages from the bronchoalveolar lavage displayed high upregulation of activation markers compared to the cells circulating in blood. Flow cytometry analysis pinpointed that COVID-19 ARDS displays a distinct cellular immunological profile in a hyperinflammatory milieu [75]. Moreover, the ratio of Th17/Treg cells and RORγt/FoxP3 were found to be increased in patients compared with the controls and in deceased patients compared with the healed ones. Using flow cytometry, it was shown that increased Th17 cells and decreased Treg cells in COVID-19 patients strongly correlates with hyperinflammation, lung damage, disease pathogenesis, and even death [76]. Moreover, innate immunity cells can be evaluated with flow cytometry. Flow cytometric analysis of peripheral blood samples identified significant morphologic and functional differences, pronounced in ARDS patients requiring ICU admission. COVID-19 patients have larger monocytes (as identified on forward scatter—FSC, side scatter—SSC) with a CD14+ CD16+ phenotype and mixed M1/M2 macrophage polarization expressing high CD80+ and CD206+. Serial monitoring using flow cytometry of inflammatory monocytes can guide prognostication and treatment of patients [77].

Cellular machinery activated to overcome the viral infection is complex, and the first autopsy of a COVID-19 patient showed accumulation of monocytes and inflammatory T cells in the lungs with low percentages of SARS-CoV-2 activated T cells [78].

An immune cellular picture of the gathered information on SARS-CoV-2 shows that CD4+ T cells, CD8+ T cells, have specific functions/kinetics; these cells interrelate with innate immunity to accomplish the efficient antiviral immune responses [79].

### 4.2. SARS-CoV-2 Immune Memory

Although we have entered the second year of the COVID-19 pandemic, the immunological memory to this infection is still a matter of data acquirement. The most facile data are coming from antibody persistence; they maintain their titer for 3–6 months [80,81,82]. However, measuring antibody titers as memory persistence is not a clear-cut indication, as antibodies would naturally decline as the organism is not in the acute phase of viral infection. As previously stated, flow cytometry is the technology that analyzes immune cells, including memory cells. Spike memory B cells could be detected 3 months postinfection [83,84,85], but we still lack information regarding up to 12–24 months’ memory cells in this infection. After 6 months postinfection, 90% positive for memory CD4+ T cells and 70% positive for memory CD8+ T cells were reported. Memory T helper cells are more abundant than cytotoxic T cells [86,87]. When asymptomatic patients were studied, it was shown that the patients have generated detectable T cell memory only slightly lower than symptomatic cases [86]. The estimated durability of CD4+ and CD8+ T cell memory of 3–5 months matches that obtained for the yellow fever virus vaccine [88], a vaccine that has very long-lasting protective immunity. Moreover, it was reported that SARS-CoV memory T cells are still active after 17 years postinfection [89]. Memory B cells specific for S, RBD, and N can be detected by flow cytometry 6 months post-COVID-19 [87,90]. Spike memory B clones were in the majority secreting IgG and only 5% IgA [87]. At 6 months postinfection, RBD memory B cells increase and synthetize higher potency, neutralizing antibodies [90,91].

When studying viral infection memory, complex measurements should be carried out, namely RBD, S antibodies, RBD-specific memory B cells, and SARS-CoV-2-specific CD4+ and CD8+ T cells. Immediately postinfection, all patients display positivity in all these immune elements, but after 6 months, variation is seen in the cellular immune compartments [92,93,94,95].

## 5. New Variants Emergence Require Tests Upgrading?

Not even one year has passed from the start of the COVID-19 pandemic when variants of concern (VOC) emerged [96]. SARS-CoV-2 is evolving, and the RBD of the S protein and the region of N protein associated with nuclear localization signals suffer positively selected amino acid replacements. Therefore, tests should be able to quickly and efficiently detect escape variants. In the initial phase, this can be achieved by sequencing, and once a variant is “of concern” (VOC), a PCR-based assay should be quickly developed and validated for worldwide tracking of the spread of the new variant. This is important not only for the infection tracking, but also for the large-scale vaccination efficacy [97]. Percipience should be exercised in the vaccine immunity escape, phenomena that can be triggered by new variants. It is known that many RNA viruses, although displaying potential variability, did not escape vaccine-induced immune response, similar to the viruses that induce measles, rubella, and many other viruses [98]. Other viruses evolve in their immunogenic regions so that they can effectively evade the host’s immune response (e.g., influenza A virus) [99]. Consequently, although SARS-CoV-2 generates a variant that escapes vaccine immunity, we do not know if this variant replicates as vigorously as the initial viral variant from which it was generated, or if it will escape vaccine immunity. However, genomic surveillance of the new variants is vital in the current pandemics [100]. The current VOCs include B.1.1.7 (Alpha), B.1.351 (Beta), B.1.617/B.1.617.2 (Delta), and P.1 (Gamma) with increased transmissibility, clinical outcome, and limitation of the diagnostic tests [101]. As this manuscript was submitted, new variants appeared, and the variant named Omicron entered the scene. This variant contains some immune-evading mutations. From 1 December 2021, Omicron was identified in 25 countries worldwide, and the list of countries detecting Omicron grows with each day [102]. Virologists expect SARS-CoV-2 to evolve predictably and add to the other respiratory viruses, but, then again, when this will occur is still not clear [103]. Until this happens, if it happens, we must cope with the existing tests and with their constant improvement.

The current standard for tracking new variants is still viral whole-genome sequencing, but the technology is costly and needs high expertise. Therefore, snapback primer-based, high-resolution melting was proposed as a less-expensive test for new variants. Published in September 2021, a newer version and less-costly test type was developed. The test can detect multiple variant lineages with over 20 important SARS-CoV-2 S mutations [104]. A nested RT-PCR assay that can detect multiple nucleotide alteration in the sequence encoding for S protein was developed for VOC identification. With this technique, in April 2021, the presence of key mutations of 20I/501Y.V1 and 20 J/501Y.V3 in the new SARS-CoV-2 variant were detected [105]. Several types of multiplex RT-qPCR targeting single nucleotide polymorphisms [106,107,108,109,110], TaqMan SARS-CoV-2 mutation panel molecular assay [111,112], combinations of novel PCR assays and genome sequencing [113], or CRISPR-Cas12-based multiplex allele-specific assay [114], just to mention a few, were able to identify VOCs, and, moreover, these tests can adapt to emerging viruses’ lineages.

Rapid antigen tests were also tested for their potency to detect VOCs such as B.1.1.7 and B.1.351; the tests were able to detect variants comparable to non-VOC strains [115]. Rapid antigen testing was compared with RT-PCR results, and, recently, it was shown that variants undetected by rapid antigen test (Panbio COVID-19, Abbott Laboratories, Jena, Germany) may be due to the T135I mutation that appeared in the N protein, therefore false-negative results should be corrected with confirmatory RT-PCR [116]. Evaluating five antigen tests, it was shown that VOCs that display up to four amino acid mutations in N were detected by the tested antigen kits [117]. In terms of rapid antigen tests performance, it is obvious that a continuous evaluation should be performed, especially concerning the evolving mutations [118]. Several POC tests were developed rapidly, with low cost, to detect infection. For example, the POC-developed test (miSHERLOCK), which is a CRISPR-based platform, was proven to be sensitive for mutations within variants B.1.1.7, B.1.351, and P.1. [119].

Another type of tests, the serological type, that assess the immune response against the infection, were evaluated in the VOCs area. When evaluating commercial anti-SARS-CoV-2 RBD antibody tests and chemiluminescent reduction neutralizing tests, it was shown that the tested sera from B.1.1.7 and B.1.351 variants-infected patients discriminated between them and healthy donors [120]. An improved, cell-based fluorescent serology assay [121] or N IgG ELISA protocol [122] were reported to have the possibility to be adapted to new spike mutated variants.

Some take-home notes regarding this huge and continuously upgrading subject focus on the fact that VOC can induce failures in standard diagnostic tests [123]. Cautious and up-to-date selection of the targets that are to be used in mutation-specific PCR is mandatory for successful detection of emerging VOCs [124].

## 6. Conclusions

In the current pandemic, a major healthcare topic is the accurate detection of a spreading infection. This detection needs to both cover the active infection and further the recovery of patients. The technologies that underlie all these complex testing methods involve two players. One is the array of standard technologies that were quickly put into use at the beginning of the current crisis, e.g., RT-PCR and ELISA, and the others are cellular tests, such as flow cytometry. From the time viewpoint, and due to the accelerated pace of this pandemic, the technological “spotlight” was taken by RT-PCR for detecting the virus and by the immune assays for detecting the generated antibodies. Left somewhere in the backstage of COVID-19 research, immune cells and, furthermore, immune memory cells, were out of this spotlight, but as we are stepping into the third year of the pandemic, and more so in the worldwide vaccination context, immune memory detection gains increasing importance. Cytometry needs, in this context, to establish the heterogeneity of COVID-19 immune memory. Specific antibodies, e.g., memory B and T cell, should be investigated as these parameters change in time during infection and postinfection. As antibody titers do not accurately indicate the appearance of memory immune cells, new data should rapidly gather on this topic. New variants can drive the panel of tests used in this pandemic to their limits. Emerging mutations can hinder diagnostic sensitivity, and postvaccine reinfections with emerging variants induce a significant economical, clinical, and public health impact. Therefore, research should further focus on improvements of diagnostic tests to accurately detect any variants of concern.

## Figures and Tables

**Table 1:** Main characteristics of molecular and antigen tests in SARS-CoV-2.

Test Type	Advantages	Disadvantages	Test Sensitivity %	Test Specificity %
Test for viral genome	Accurate tests, identifies mutations in the virus, it tracks disease spread.	Does not detect viral load, does not detect dynamics of infection or the history of prior infection.	86.1%	95.8%
Test for viral antigen	Detects proteins on the viral particle surface.	Less sensitive than molecular tests and often a molecular test need to confirm the positive result.	61.7%	98.2%
	Faster than molecular tests, less expensive, applicable to large number of samples.			
